# Electronic intrapartum fetal monitoring: a systematic review of international clinical practice guidelines

**DOI:** 10.1016/j.xagr.2021.100008

**Published:** 2021-03-06

**Authors:** Manoj Mohan, Joohi Ramawat, Gene La Monica, Pradeep Jayaram, Sherif Abdel Fattah, Jonathan Learmont, Corinna Bryan, Safia Zaoui, Abdul Kareem Pullattayil, Justin Konje, Stephen Lindow

**Affiliations:** a Sidra Medicine, Doha, Qatar (Drs Mohan and Learmont and Mr Pullattayil and Prof Konje); b Weill Cornell Medicine, Ar-Rayyan, Qatar (Dr Mohan); c Al Wakra Hospital, Hamad Medical Corporation, Doha, Qatar (Drs Ramawat and Zaoui); d Division of Maternal-Fetal Medicine, Hospital Corporation of America Houston Healthcare Southeast, Pasadena, TX (Dr Monica La Monica); e Sidra Medical and Research Center, Doha, Qatar (Dr Jayaram); f Department of Obstetrics and Maternal-Fetal Medicine, Southmead Hospital, United Kingdom (Dr Fattah); g Department of Obstetrics and Maternal-Fetal Medicine, Evangelischen Krankenhaus Hamm Medical Center, Hamm, Germany (Dr Bryan); h Coombe Women and Infants University Hospital, Dublin, Ireland (Prof Lindow).

**Keywords:** electronic fetal monitoring, cardiotocography, clinical practice guidelines, obstetrics

## Abstract

**BACKGROUND:**

Electronic fetal monitoring or fetal assessment using a cardiotocograph is currently the most commonly employed tool for intrapartum surveillance. Furthermore, there are numerous guidelines informing best practice worldwide.

**OBJECTIVE:**

This systematic review aimed to compare and appraise all available practice guidelines on intrapartum electronic fetal monitoring to describe the similarities and variations in recommendations.

**STUDY DESIGN:**

A systematic protocol was developed per Preferred Reporting Item for Systematic Review and Meta-Analysis Protocols. A total of 4 independent reviewers were involved with independent searches and quality assessment using the Appraisal of Guidelines for Research and Evaluation Instrument for guideline quality reporting.

**RESULTS:**

Overall, 7 international practice guidelines were included in this systematic review. Appraisal of Guidelines for Research and Evaluation Instrument showed higher scores for scope and purpose and for clarity of presentation; however, the overall assessment varied between 25% and 89%. When individual characteristics of electronic fetal monitoring or cardiotocograph were compared, all guidelines and guidance were essentially trying to describe the characters similarly, with critical differences described in the full article.

**CONCLUSION:**

In the context of globalization, a uniform approach for defining terminology, classifying characters and similar interpretation of results is needed for electronic fetal monitoring. Therefore, we should consider a unified, simple, logistically approved, and acceptable guideline, which is probably accepted worldwide.

**AJOG MFM at a Glance**

**Why was this study conducted?**

To study the variations and similarities of international practice guidelines on electronic intrapartum fetal monitoring.

**Key findings**

All guidelines are essentially trying to describe the characteristics similarly with some essential variations identified and described.

**What does this add to what is known?**

The pieces of information for future guideline developments regarding a unified global approach for interpreting and standardizing electronic intrapartum monitoring.

## Introduction

Electronic fetal monitoring (EFM) or cardiotocography (CTG) is the current mainstay of intrapartum fetal monitoring worldwide. At its inception, there were no objective data or evidence to support its use, and there was certainly no randomized trial. A recent systematic review^1^ concluded that the use of CTG or EFM made no difference to clinical outcomes, such as cesarean delivery or instrumental deliveries. Several guidelines have been developed by institutions or international bodies to govern practice in several hospitals. Although there are agreements with these guidelines, there are also differences and variations. For example, fetal heart tracing is classified as “abnormal” attribute variations.^2^^,^^3^

CTG or EFM has significant interobserver variability in interpretation.^4^ Particularly, the American College of Obstetricians and Gynecologists (ACOG) guideline shows the highest interobserver agreement for category II tracings and the lowest interobserver agreement for category I and III tracings. In addition, the variations for the prediction of acidemia varies between the ACOG and International Federation of Gynecology and Obstetrics (FIGO) guidelines. Furthermore, there were low but fair interobserver variations when comparing the French guideline with the FIGO guideline. The choice between these guidelines could have an impact on first cesarean delivery decision.^5^

Data from the last 60 minutes before delivery have shown that it is difficult to accurately estimate fetal acidemia.^6^ This could be related to (1) the inter- and intraobserver differences in interpretation of EFM of CTG, (2) the actual definition of fetal heart rate features used for the interpretation, and (3) a sustained, systematic error in the build of all guidelines across CTG or EFM. Consequently, there have been attempts to move away from pattern recognition to physiological approach at CTG interpretation, possibly trying to reduce the variations.^7^

It is more likely that the use of additional high-tech advances, such as remote wearable technology^8^ and computer-analyzed technologies^9^ and artificial intelligence, may likely replace the human interphase or interpretation for investigations, such as CTG or EFM. However, not having a defined or common agreement is likely to compromise this future perspective for any progress in EFM.

In summary, several guidelines are used worldwide with a background understanding of interobserver variance and observed variations, such as prediction of acidemia among international practice guidelines. Newer technologies are arising with the use of these basic principles. Considering all these factors together, there is currently no study available to compare all the international practice guidelines to understand the commonalities and differences and to improve the further standard of care. Therefore, we presented a systematic review of CTG or EFM guidelines, explicitly looking at recommendations that are particularly focused on how to describe CTG or EFM.

## Methods

### Sources

A priori protocol was defined per Preferred Reporting Item for Systematic Review and Meta-Analysis Protocols (PRISMA-P),^10^ which included defined objectives, criteria for guideline selection, and approach to assessing outcomes. All reviewers had undertaken relevant training on Appraisal of Guidelines for Research and Evaluation (AGREE) on the AGREE Enterprise website^11^ and had previous experience in local and international guideline appraisals. The protocol developed was registered on the International Prospective Register of Systematic Reviews (CRD42018085085). The review was reported in accordance with the PRISMA statement.^12^

The initial literature search was performed on June 27, 2019, by M.M. and J.R. and repeated by our librarian A.P., and the search was further updated on July 19, 2020, and the contents of the publication updated with the final revised search. Electronic databases included in the searches were PubMed, Embase, Cochrane Central Register of Controlled Trials, Scopus, and Web of Science. Furthermore, hand search was performed. The search words included electronic fetal monitoring, cardiotocography, pregnancy, obstetrics, and labo*. In addition, we specifically searched for clinical practice guideline-producing bodies, including the National Guideline Clearinghouse, the National Institute for Health and Care Excellence, FIGO, the Scottish Intercollegiate Guidelines Network, the ACOG, the Royal College of Obstetricians and Gynaecologists, the Society of Obstetricians and Gynaecologists of Canada, the German Society of Gynecology and Obstetrics, and the Royal Australian and New Zealand College of Obstetricians and Gynaecologists. Other international bodies, including the World Health Organization and international societies of obstetrics and gynecology from Belgium, France, and the rest of the world, were also searched to completely include all internationally developed and organizationally affiliated accepted practice guidelines (Figure).

**Figure:**
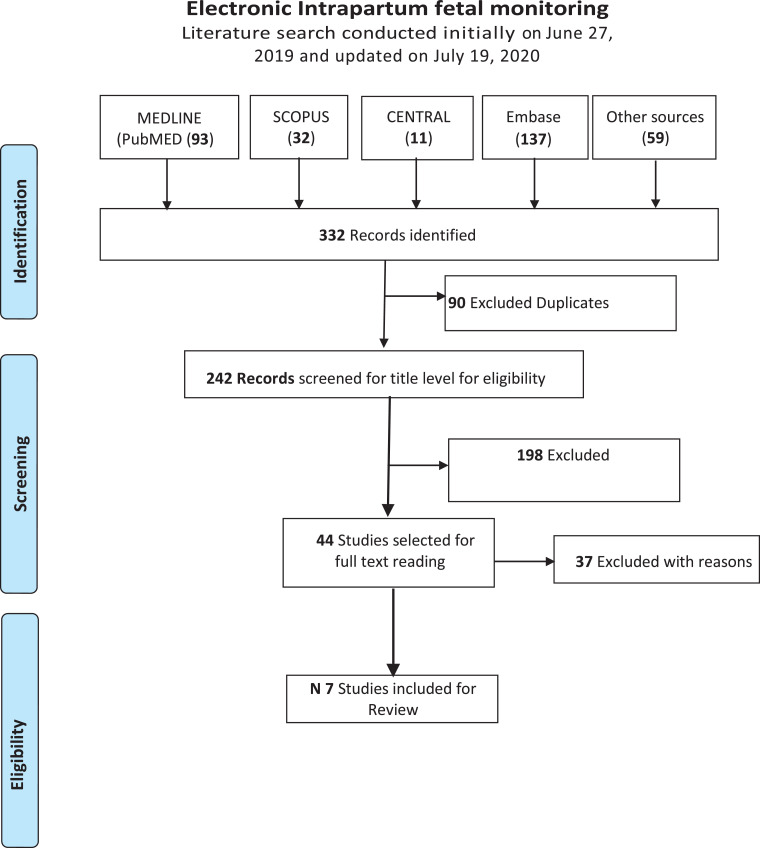
PRISMA flow diagram Adapted from Moher et al.^12^ *PRISMA*, Preferred Reporting Items for Systematic Reviews and Meta-Analyses. *Mohan. Electronic intrapartum fetal monitoring. Am J Obstet Gynecol Glob Rep 2021*.

### Guideline selection

All titles and abstracts were screened on the basis of the selection criteria by M.M. and J.R. independently to select the relevant guidelines per the protocol. Furthermore, all titles and abstracts were rescreened and compared for validity by G.L. and P.J. independently. For uncertain titles and abstracts, a group consensus was obtained. All 4 primary reviewers independently screened the full contents of the selected guidelines and then assessed them using the AGREE II platform electronically to collate the published results. The AGREE II scoring by independent reviewers only had minor expected variations, which was summated using the AGREE II software, and therefore did not have any major discrepancy.

### Selection criteria

#### Inclusion

Published clinical practice guidelines of national or international bodies were included if the (1) recommended guidelines included the mode of identifying all features of EFM or CTG and provided a recommendation or actions necessary to manage the identified features appropriately and (2) if the recommendation could be part of an independent EFM or CTG intrapartum guideline or could be part of the main guideline of pregnancy, which includes intrapartum care with EFM or CTG.

#### Exclusion

The following guidelines were excluded from the review:

1. Regional or institutional guidelines

2. Guidelines more than 15 years old with current or updated versions

3. Scientific papers, including committee opinions, scientific impact papers, reviews, commentaries, and journal club articles

4. Unreferenced guidelines or recom-mendations

#### Recommendations studied

The following recommendations were studied:

1. Baseline rate and variations

2. Variability and variations

3. Deceleration and variations

4. Accelerations and variations

5. Recommendations for interpretations and actions

6. Recommendation on intrapartum fetal blood sampling

In addition, general characteristics, including the status of publication, type of organization, and type or grade of recommendations, were examined. Furthermore, 2 guidelines were excluded—a Japanese guideline^13^ because it was based on fetal heart rate pattern only and therefore could not be compared with other guidelines and a Polish guideline^14^ because it was as an adaptation of the ACOG guideline (it was excluded to avoid duplication).

### Quality assessment

A total of 4 reviewers (M.M., J.R., G.L., and P.J.) underwent and completed appropriate training in the use of the quality assessment instrument (AGREE II) and then used the instrument independently.^11^ The distribution and collation were performed by M.M. and were reviewed by S.L. Each of the 23 items in all 6 domains was assessed on a 7-point scale. A score of 7 indicated exceptional quality reporting and that all the criteria and considerations articulated by AGREE II were met. A score of 1 indicated an absence of information or that the concept is poorly reported. A score between 6 and 2 indicated that the quality of reporting did not fully or only partially complied with AGREE II. The percentage of the maximum score in each domain was calculated. The final score calculated by M.M. and reviewed by S.L. was then agreed for consensus by all authors.

## Results

This systematic review using AGREE II included 7 guidelines^2^^,^^3^^,^^15^, ^16^, ^17^, ^18^, ^19^ published from 2007 to 2020, and 5 of these guidelines have been recently updated.^2^^,^^3^^,^^15^, ^16^, ^17^ The general characteristics of the guidelines are represented in Table 1.

**Table 1:** General characteristics of guidelines

	Characteristic	SOGC	ACOG	NICE	FIGO	RANZCOG	DGGG	CNGOF
Status								
	Published year	2007	2005	2014	2015	2001	2005	2007
	Updated	2020	2009	2017		2014	2014	
	Separate intrapartum fetal monitoring or surveillance guideline	Yes	Yes	No	Yes	Yes	Yes	Yes
	Part of a main guideline	No	No	Yes	No	No	No	No
Organizational								
	Professional body	Yes	Yes	Yes	Yes	Yes	Yes	Yes
	Governmental	No	No	No	No	No	No	Yes
Recommendations								
	Evidence level stated	Yes	Yes	Yes	No	Yes	Yes	No
	Recommendation GRADE or equivalent stated	Yes	Yes	Yes	No	Yes	Yes	Yes

*ACOG*, American College of Obstetricians and Gynecologists; *CNGOF*, National College of French Gynaecologists and Obstetricians; *DGGG*, German Society and Gynecology and Obstetrics; *FIGO*, Federation of Gynecology and Obstetrics; *NICE*, National Institute for Health and Care Excellence; *RANZCOG*, Royal Australian and New Zealand College of Obstetricians and Gynaecologists; *SOGC*, Society of Obstetricians and Gynaecologists of Canada.

Mohan. Electronic intrapartum fetal monitoring. Am J Obstet Gynecol Glob Rep 2021.

We specifically looked into the recommendations per our inclusion criteria, and apart from fetal blood sampling, all guidelines had reported on all the other recommendations. The guideline recommendations are provided in Table 2.

**Table 2:** Guideline recommendations

Recommendation included	SOGC	ACOG	NICE	FIGO	RANZCOG	DGGG	CNGOF
Describe features of CTG	Yes	Yes	Yes	Yes	Yes	Yes	Yes
Interpretation or action Normal or reassuring (category 1)	Yes	Yes	Yes	Yes	Yes	Yes	Yes
Interpretation or action Suspicious, atypical, or nonreassuring (category 2)	Yes	Yes	Yes	Yes	Yes	Yes	Yes
Interpretation or action Abnormal or pathologic (category 3)	Yes	Yes	Yes	Yes	Yes	Yes	Yes
Interpretation of fetal blood sampling	Yes	No	Yes	No	No	Yes	No

*ACOG*, American College of Obstetricians and Gynecologists; *CNGOF*, National College of French Gynaecologists and Obstetricians; *CTG*, Cardiotocography; *DGGG*, German Society and Gynecology and Obstetrics; *FIGO*, Federation of Gynecology and Obstetrics; *NICE*, National Institute for Health and Care Excellence; *RANZCOG*, Royal Australian and New Zealand College of Obstetricians and Gynaecologists; *SOGC*, Society of Obstetricians and Gynaecologists of Canada.

Mohan. Electronic intrapartum fetal monitoring. Am J Obstet Gynecol Glob Rep 2021.

The AGREE II scores showed similarities and variations in each guideline or guidance produced. The scope and purpose (63%–97%) of the guidelines and presentation clarity (60%–91%) were high. However, the overall assessment varied between 25% and 89%, and the variation was predominantly because of the lack of information in the published guidelines with respect to rigor development (28%–90%) and editorial independence (19%–67%) (Table 3).

**Table 3:** AGREE II scores

	SOGC	ACOG	NICE	FIGO	RANZCOG	DGGG	CNGOF
AGREE II domains							
1. Scope and purpose	93%	78%	64%	63%	97%	92%	88%
2. Stakeholder involvement	69%	51%	75%	36%	71%	57%	56%
3. Rigor of development	82%	58%	80%	28%	90%	59%	46%
4. Clarity of presentation	91%	82%	86%	60%	86%	74%	67%
5. Applicability	57%	83%	83%	25%	73%	51%	43%
6. Editorial independence	64%	29%	67%	19%	94%	48%	35%
Overall assessment	89%	63%	79%	25%	88%	63%	50%

*ACOG*, American College of Obstetricians and Gynecologists; *AGREE*, Appraisal of Guidelines for Research and Evaluation; *CNGOF*, National College of French Gynaecologists and Obstetricians; *DGGG*, German Society and Gynecology and Obstetrics; *FIGO*, Federation of Gynecology and Obstetrics; *NICE*, National Institute for Health and Care Excellence; *RANZCOG*, Royal Australian and New Zealand College of Obstetricians and Gynaecologists; *SOGC*, Society of Obstetricians and Gynaecologists of Canada.

Mohan. Electronic intrapartum fetal monitoring. Am J Obstet Gynecol Glob Rep 2021.

## Discussion

This systematic review showed much higher scores for the scope and purpose of the 7 guidelines, suggesting that most developments of guidelines focused on the overall objectives of the guidelines. The guidelines specifically describe health questions, such as the need for CTG or EFM in clinical practice. The clarity of presentation is specific and unambiguous, and all guidelines provide key recommendations for clinical practice, which are highlighted in the guidelines and easy to follow.

The overall assessment varied between 25% and 89%, predominantly because of the unavailability of information per AGREE II. Most information could not be extracted from the published guidelines, some of which included the systematic search methods not described, the criteria for selecting evidence not described in the guideline, and the strength and limitation of evidence not well described.

In addition, editorial independence varied (19%–67%) because some of the guidelines did not address the views of the funding body that influence the content of the guideline and because competing interests of the guideline development group members were not addressed.

This study showed similarities and differences in various national and international guidelines in the interpretation of CTG (Table 4, Table 5, Table 6, Table 7).

**Table 4:** Cardiotocography compared

Variable	SOGC (Canadian)	ACOG (American)	NICE (British)	FIGO	RANZCOG (Australian)	GOGS (German)	CNGOF (French)
Baseline Normal or reassuring (category 1)	110–160 bpm	110–160 bpm	100–160 bpm	110–160 bpm	110–160 bpm	110–160 bpm	110–160 bpm
Baseline bradycardia Suspicious, atypical, or nonreassuring (category 2)	100–110 bpm	<110 bpm and no absent variability		<110 bpm for >10 min	100–109 bpm	100–109 bpm	100–110 bpm (L) 90–100 bpm (M) <90 bpm (H)
Baseline tachycardia Suspicious, atypical, or nonreassuring (category 2)	>160 bpm for >30 min to <80 min Rising baseline	>160 bpm	161–180 bpm	>160 bpm for >10 min		161–180 bpm (slight) >180 bpm (severe)	160–180 bpm (L) >180 bpm (H)
Baseline bradycardia Abnormal or pathologic (category 3)	<100 bpm >160 bpm for >80 m Erratic baseline	<110 b	<100 bpm >180 bpm	<100 bpm	>160 bpm	100–109 bpm (slight) <100 bpm (severe)	<90 bpm
			<100 bpm for ≥3 min		<100 bpm for ≥5 min (sinusoidal)	Sinusoidal (at least 10 min)	
Variability Normal or reassuring (category 1)	6–25 bpm for <5 min to <40 min	Moderate	>5 bpm	5–25 bpm	6–25 bpm <3 bpm	≥5 bpm	6–25 bpm
Variability Suspicious, atypical, or nonreassuring (category 2)	>5 bpm for 40–80 min	<3 bpm or <5 bpm for >25 min	<5 bpm for 30–90 min		Reduced 3–5 bpm	<5 bpm for ≥40 min but ≤90 min or >25 bpm	3–5 bpm for <40 min (L)
Variability Abnormal or pathologic (category 3)	≤5 bpm for >80 min >25 bpm for >10 min	Absent	<5 bpm for ≥90 min	<5 bpm for >50 min >25 bpm for >30 min	Absent (<3 bpm)	<5 bpm for ≥90 min	3–5 bpm for >40 min (M) 3–5 bm for <40 min
	Sinusoidal	Sinusoidal		Sinusoidal, >30 min			Sinusoidal (H)

L indicates low risk of acidosis; M indicates moderate risk of acidosis; and H indicates high risk of acidosis

*ACOG*, American College of Obstetricians and Gynecologists; *CNGOF*, National College of French Gynaecologists and Obstetricians; *DGGG*, German Society and Gynecology and Obstetrics; *FIGO*, Federation of Gynecology and Obstetrics; *NICE*, National Institute for Health and Care Excellence; *RANZCOG*, Royal Australian and New Zealand College of Obstetricians and Gynaecologists; *SOGC*, Society of Obstetricians and Gynaecologists of Canada.

Color codes

Mohan. Electronic intrapartum fetal monitoring. Am J Obstet Gynecol Glob Rep 2021.

**Table 5:** Cardiotocography compared

Variable	SOGC (Canadian)	ACOG (American)	NICE (British)	FIGO	RANZCOG (Australian)	GOGS (German)	CNGOF (French)
Deceleration Normal or reassuring (category 1)	None or nonrepetitive uncomplicated variable decelerations or early decelerations	Late or variable deceleration absent Early deceleration present or absent	None or early	No repetitive decelerations (ie, in less than 50% contraction)	None	None	Absent
Deceleration Suspicious, atypical, or nonreassuring (category 2)	Repetitive uncomplicated variables or nonrepetitive complicated variables or intermittent late deceleration or single prolonged ≥2 min but <3 min	Periodic or episodic accompanied by minimal or moderate baseline variability or recurrent late with mod variability	Variable (≤60 bpm for ≤60 sec) for >90 min Variable (>60 bpm for >60 sec) for ≤30 min Late ≤30 bpm (all with over 50% of contractions)	Lacking 1 feature of normality but with no pathologic features	Early Variable without complicating feature	Early or variable deceleration or prolonged (deceleration <3 min)	Early Variable (<60 sec and depth <60 bpm or prolonged but <3 min) (L)
Deceleration Abnormal, or pathologic (category 3)	Repetitive complicated variables or recurrent late decelerations	Absent variability with recurrent late or variable or bradycardia	Nonreassuring variable after 30 min after conservative measures Late deceleration present for 30 min do not improve with conservative measures	Repetitive (more than 50% of contractions) Late or prolonged decelerations >30 min or 20 min if reduced variability	Complicated variable Late or prolonged complicated variable deceleration with reduced or absent variability Late deceleration with reduced or absent variability	Late or atypical variable decelerations	Variable (<60 sec and depth >60 bpm) Prolonged >3 min (M) Repeated late variable (>60 sec) (H)
	Single prolonged >3 min but <10 min	Prolonged <10 min	Bradycardia or prolonged >3 min	1 prolonged >5 min	Prolonged >5 min	Single prolonged >3 min	Prolonged >3 min (H)

L indicates low risk of acidosis; M indicates moderate risk of acidosis; and H indicates high risk of acidosis

*ACOG*, American College of Obstetricians and Gynecologists; *CNGOF*, National College of French Gynaecologists and Obstetricians; *DGGG*, German Society and Gynecology and Obstetrics; *FIGO*, Federation of Gynecology and Obstetrics; *NICE*, National Institute for Health and Care Excellence; *RANZCOG*, Royal Australian and New Zealand College of Obstetricians and Gynaecologists; *SOGC*, Society of Obstetricians and Gynaecologists of Canada.

Color codes

Mohan. Electronic intrapartum fetal monitoring. Am J Obstet Gynecol Glob Rep 2021.

**Table 6:** Cardiotocography compared

Variable	SOGC (Canadian)	ACOG (American)	NICE (British)	FIGO	RANZCOG (Australian)	GOGS (German)	CNGOF (French)
Acceleration Normal or reassuring (category 1)	Spontaneous acceleration but not required or scalp stimulation	Acceleration present or absent		Abrupt	15 bpm for 15 sex	2 bpm in 20 min	Present
Acceleration Suspicious, atypical, or nonreassuring (category 2)	Absence of acceleration with scalp stimulation	Absence of Acceleration after stimulation				Periodic with every contractions	Present or absent
Acceleration Abnormal or pathologic (category 3)	Usually absent (if present, do not change classification)					None >40 min (unclear significance)	Present or absent
Interpretation or action Normal or reassuring (category 1)	No evidence of fetal compromise	Strongly predictive of normal acid base status May be monitored in routine manner No specific action	All 3 features are normal or reassuring normal CTG, no nonreassuring or abnormal features, healthy fetus Continue normal care	Fetus with no hypoxia or acidosis No intervention necessary to improve fetal oxygen state	Low probability of fetal compromise	All 4 evaluations are normal Action: none	Continuous CTG monitoring
Interpretation or action suspicious, atypical, or nonreassuring (category 2: low risk of acidosis)	Physiological response	Requires evaluation May require ancillary test of fetal well-being like PH or lactate	1 nonreassuring feature and 2 normal or reassuring features Combination features with increased risk of fetal acidosis Action: assess conservative measures	Fetus with low probability of hypoxia or acidosis Correct reversible causes, close monitoring or additional methods to evaluate fetal oxygenation	Unlikely to be associated with fetal compromise when occurring in isolation	At least 1 evaluation criteria suspected and all other normal Action: conservative	Resuscitation, if no improvement for further actions
Interpretation or action Abnormal or pathologic (category 3: moderate or High risk of acidosis)	Possible fetal compromise	Abnormal fetal acid base status Requires prompt evaluation, include resuscitative measures or delivery	1 abnormal feature or 2 nonreassuring features Abnormal and needs conservative measure with further testing Assess conservative measures Offer to take an FBS (for lactate or pH ) or delivery	Fetus with high probability of hypoxia or acidosis Action to correct acidosis or delivery	Associated with significant fetal compromise and need further action Identify reversible cause and initiation of appropriate or urgent delivery	At least 1 evaluation criterion pathologic or 2 or more suspicious Action: conservatively and invasive	Immediate further actions if high risk of acidosis or immediate fetal extraction if further action not indicated

*ACOG*, American College of Obstetricians and Gynecologists; *CNGOF*, National College of French Gynaecologists and Obstetricians; *CTG*, cardiotocography. *DGGG*, German Society and Gynecology and Obstetrics; *FBS*, fasting blood sugar; *FIGO*, Federation of Gynecology and Obstetrics; *NICE*, National Institute for Health and Care Excellence; *RANZCOG*, Royal Australian and New Zealand College of Obstetricians and Gynaecologists; *SOGC*, Society of Obstetricians and Gynaecologists of Canada.

Color codes

Mohan. Electronic intrapartum fetal monitoring. Am J Obstet Gynecol Glob Rep 2021.

**Table 7:** Cardiotocography compared

Variable	SOGC (Canadian)	ACOG (American)	NICE (British)	FIGO	RANZCOG (Australian)	GOGS (German)	CNGOF (French)
Fetal blood sampling	pH≥7.25 Lactate<4.2 mmol/L Normal Repeat in 30 min if abnormality persists		Normal Lactate≤4.1 mmol/L pH≥7.25			pH≥7.25—repeated after 30 min if abnormality persists	
	pH=7.21–7.24 Lactate=4.2–4.8 mmol/L Borderline Repeat within 30 min or consider delivery if significant fall in pH or rise in lactate		Borderline Lactate=4.2–4.8 mmol/L pH=7.21–7.24			pH=7.21–7.24—repeat in 30 min or rapid delivery	
	pH≤7.20 Lactate>4.8 mmol/L Abnormal Delivery indicated		Abnormal Lactate≥4.9 mmol/L pH≤7.20			pH≤7.20 pCO_2_>65 mm Hg BE greater than −9.8, quick delivery indicated	

*ACOG*, American College of Obstetricians and Gynecologists; *CNGOF*, National College of French Gynaecologists and Obstetricians; *DGGG*, German Society and Gynecology and Obstetrics; *FIGO*, Federation of Gynecology and Obstetrics; *NICE*, National Institute for Health and Care Excellence; *RANZCOG*, Royal Australian and New Zealand College of Obstetricians and Gynaecologists; *SOGC*, Society of Obstetricians and Gynaecologists of Canada.

Color codes

Mohan. Electronic intrapartum fetal monitoring. Am J Obstet Gynecol Glob Rep 2021.

The areas of dissimilarities where we believe there could be discussions on standardization included defining the baseline heart rate, bradycardia, and/or duration of prolonged bradycardia. There needs to be a unified approach, as these variations could be associated with neonatal outcomes secondary to nonstandardization.

There is a need to form a consensus about whether or not to include a sinusoidal pattern in the guidelines or whether the sinusoidal pattern belongs to the baseline rate or variability group. In addition, some guidelines describe prolonged deceleration in the baseline, whereas others do in the deceleration category. This may not necessarily affect the clinical management and outcome of the monitored neonates but may confuse those delivering clinical practice worldwide as to why the parameters are in different groups.

EFM or CTG is known to have an interobserver agreement but with variation,^4^ and a Cochrane systematic review^1^ showed that CTG monitoring in labor reduced the rate of neonatal seizures, with no clear difference in cerebral palsy, infant mortality, or neonatal well-being. However, CTG use was associated with increased maternal morbidity, including increased cesarean delivery interventions and instrumental deliveries. All of these anomalies could be related to variations in interpretation.

Single characters of EFM or CTG studied, such as the accepted baseline rate, have addressed differences in the baseline rates and suggested accepted standards.^20^ Furthermore, we should objectively assess other EFM or CTG characters and whether they should be used in standard guidelines.

Similarly, the outcomes from the recent INFANT and follow-up study^21^^,^^22^ suggested that even the use of “decision support software” in intrapartum fetal monitoring played no role in outcomes compared with CTG alone. The study excluded the associated human factors in the study group and showed no difference in the outcome. This finding could be attributed to the fact that the clinical guidance on EFM or CTG monitoring contains some conflicting factors that might lead to different interpretations and therefore sometimes fail to identify when to deliver.

Therefore, this commonly used tool for intrapartum fetal monitoring needs much more rigorous testing to determine its diagnostic accuracy. There need to be accurately defined and unified standards. There is a requirement for more studies on individual components of EFM of CTG, for example, the baseline rate study.^20^ Overall, CTG requires further study and analysis to fully understand how an accepted consensus guideline can be reached.

Based on these findings and implications, we would recommend a “world consensus CTG guideline.” Representatives or groups with EFM or CTG expertise should be identified worldwide and brought together to construct a “consensus international guideline.”

## Conclusion

This study showed that the 7 international practice guidelines reviewed had very well-defined scopes describing their development and were presented very well with clarity. However, guideline development committees should also attempt to describe the rigorous development process, such as a summary on evidence search, criteria for selecting the obtained evidence, and the strengths and limitations. All these should be provided either in the guideline or as a supplement. The guideline development committee should always declare their conflict of interest to offer editorial independence and broader acceptance of understanding without any conflicts.

This study demonstrated that guidelines worldwide generally convey the same principles behind interpreting intrapartum EFM or CTG, but practice differences exist.

In the context of globalization, a uniform approach by all the guideline-producing bodies to provide a single, simple, logistically approvable guideline or a synchronous approach by international guideline-developing committees to work together to minimize variation is recommended. Using agreed terminology, in particular, there should be a consensus description of bradycardia and prolonged deceleration as these may affect the outcome of sentinel hypoxic events.

In addition, the deficiencies of EFM of CTG need more research along with consensus reporting or guideline approach worldwide. In the context of globalization, an internationally accepted unified guideline is a practical solution; however, this means that all guideline-producing bodies or societies need to think about how best to produce this.

## Appendix Supplementary materials

Image, application 1

## References

[1] Alfirevic Z, Devane D, Gyte GM, Cuthbert A. (2017). Continuous cardiotocography (CTG) as a form of electronic fetal monitoring (EFM) for fetal assessment during labour. Cochrane Database Syst Rev.

[2] Dore S, Ehman W. (2020). No. 396-fetal health surveillance: intrapartum consensus guideline. J Obstet Gynaecol Can.

[3] NICE Clinical Guideline 190, NICE (National Institute for Health and Care Excellence). 2017. Available at: https://www.nice.org.uk/guidance/cg190/resources/interpretation-of-cardiotocograph-traces-pdf-248732173. Accessed March 25, 2021.

[4] Santo S, Ayres-de-Campos D, Costa-Santos C (2017). Agreement and accuracy using the FIGO, ACOG and NICE cardiotocography interpretation guidelines. Acta Obstet Gynecol Scand.

[5] Vejux N, Ledu R, D'ercole C, Piechon L, Loundou A, Bretelle F (2017). Guideline choice for CTG analysis influences first caesarean decision. J Matern Fetal Neonatal Med.

[6] Kundu S, Kuehnle E, Schippert C, von Ehr J, Hillemanns P, Staboulidou I. (2017). Estimation of neonatal outcome artery pH value according to CTG interpretation of the last 60 min before delivery: a retrospective study. Can the outcome pH value be predicted?. Arch Gynecol Obstet.

[7] Chandraharan E (2018). https://physiological-ctg.com/guideline.html.

[8] Signorini MG, Fanelli A, Magenes G. (2014). Monitoring fetal heart rate during pregnancy: contributions from advanced signal processing and wearable technology. Comput Math Methods Med.

[9] Ayres-de-Campos D, Rei M, Nunes I, Sousa P, Bernardes J. (2017). SisPorto 4.0 - computer analysis following the 2015 FIGO Guidelines for intrapartum fetal monitoring. J Matern Fetal Neonatal Med.

[10] Moher D, Shamseer L, Clarke M (2015). Preferred reporting items for systematic review and meta-analysis protocols (PRISMA-P) 2015 statement. Syst Rev.

[11] Brouwers MC, Kho ME, Browman GP (2010). AGREE II: advancing guideline development, reporting and evaluation in health care. CMAJ.

[12] Moher D, Liberati A, Tetzlaff J, Altman DG, Group PRISMA (2009). Preferred Reporting Items for Systematic Reviews and Meta-Analyses: the PRISMA statement. PLoS Med.

[13] Okai T, Ikeda T, Kawarabayashi T (2010). Intrapartum management guidelines based on fetal heart rate pattern classification. J Obstet Gynaecol Res.

[14] Polish Gynecological Society (2014). [Recommendations of the Polish Gynecological Society concerning application of cardiotocography in obstetrics]. Ginekol Pol.

[15] Viswanatha RK, Talaulikar VS, Arulkumaran S. (2017). Intrapartum fetal surveillance. Obstet Gynaecol Reprod Med.

[16] (2009). ACOG Practice Bulletin no. 106: intrapartum fetal heart rate monitoring: nomenclature, interpretation, and general management principles. Obstet Gynecol.

[17] German Society of Gynecology and Obstetrics (DGGG), Maternal-Fetal Medicine Study Group (AGMFM), German Society of Prenatal Medicine and Obstetrics (DGPGM), German Society of Perinatal Medicine (DGPM) (2014). S1-guideline on the use of CTG during pregnancy and labor: long version - AWMF Registry No. 015/036. Geburtshilfe Frauenheilkd.

[18] Ayres-de-campos D, Spong CY, Chandraharan E (2015). FIGO Intrapartum Fetal Monitoring Expert Consensus Panel. Figo consensus guidelines on intrapartum fetal monitoring: cardiotocography. Int J Gynecol Obstet.

[19] Martin A. (2008). [Fetal heart rate during labor: definitions and interpretation]. J Gynecol Obstet Biol Reprod (Paris).

[20] Pildner von Steinburg S, Boulesteix A-L, Lederer C (2013). What is the “normal” fetal heart rate?. PeerJ.

[21] INFANT Collaborative Group (2017). Computerised interpretation of fetal heart rate during labour (INFANT): a randomised controlled trial. Lancet.

[22] Steer PJ, Kovar I, McKenzie C, Griffin M, Linsell L. (2019). Computerised analysis of intrapartum fetal heart rate patterns and adverse outcomes in the INFANT trial. BJOG.

