# Supplementary material for: Electronic intrapartum fetal monitoring: a systematic review of international clinical practice guidelines
Source: AJOG Glob Rep. 2021 Mar 6;1(2):100008. doi: 10.1016/j.xagr.2021.100008 (PMC9563206; doi:10.1016/j.xagr.2021.100008)
Supplement: Supplementary file 1 [file mmc1.pdf]

<u>Search Strategy</u>

Electronic Intrapartum fetal monitoring Literature search conducted initially on June 27, 2019 and updated on July 19, 2020

Database: PubMed, first search on June 27, 2019 and update on July 19, 2020

#	Query	Items found
	(((((("foetal monitoring"[All Fields] OR "fetal monitoring"[MeSH Terms]) OR ("fetal"[All Fields] AND "monitoring"[All Fields])) OR "fetal monitoring"[All Fields]) OR ("cardiotocography"[MeSH Terms] OR "cardiotocography"[All Fields])) AND ((((((("pregnancy"[MeSH Terms] OR "pregnancy"[All Fields]) OR "pregnancies"[All Fields]) OR "pregnancy s"[All Fields]) OR ((((((((((((((((("labor s"[All Fields] OR "labored"[All Fields]) OR "laborer"[All Fields]) OR "laborer s"[All Fields]) OR "laborers"[All Fields]) OR "laboring"[All Fields]) OR "labors"[All Fields]) OR "labour"[All Fields]) OR "work"[MeSH Terms]) OR "work"[All Fields]) OR "labor"[All Fields]) OR "labor, obstetric"[MeSH Terms]) OR ("labor"[All Fields] AND "obstetric"[All Fields])) OR "obstetric labor"[All Fields]) OR "laboured"[All Fields]) OR "labourer"[All Fields]) OR "labourers"[All Fields]) OR "labouring"[All Fields]) OR "labours"[All Fields])) OR (((("deliveries"[All Fields] OR "delivery, obstetric"[MeSH Terms]) OR ("delivery"[All Fields] AND "obstetric"[All Fields])) OR "obstetric delivery"[All Fields]) OR "delivery"[All Fields])) OR ((((((("child"[MeSH Terms] OR "child"[All Fields]) OR "children"[All Fields]) OR "child s"[All Fields]) OR "children s"[All Fields]) OR "childrens"[All Fields]) OR "childs"[All Fields]) AND (((((("birth s"[All Fields] OR "birthed"[All Fields]) OR "birthing"[All Fields]) OR "parturition"[MeSH Terms]) OR "parturition"[All Fields]) OR "birth"[All Fields]) OR "births"[All Fields]))) OR (((((("birth s"[All Fields] OR "birthed"[All Fields]) OR "birthing"[All Fields]) OR "parturition"[MeSH Terms]) OR "parturition"[All Fields]) OR "birth"[All Fields]) OR "births"[All Fields]))) AND (((((("pathway"[All Fields] OR "pathway s"[All Fields]) OR "pathways"[All Fields]) OR ((((((("algorithm s"[All Fields] OR "algorithmic"[All Fields]) OR "algorithmically"[All Fields]) OR "algorithmics"[All Fields]) OR "algorithmization"[All Fields]) OR "algorithms"[MeSH Terms]) OR "algorithms"[All Fields]) OR "algorithm"[All Fields])) OR (((("clinical protocols"[MeSH Terms] OR ("clinical"[All Fields] AND "protocols"[All Fields])) OR "clinical protocols"[All Fields]) OR ("clinical"[All Fields] AND "protocol"[All Fields])) OR "clinical protocol"[All Fields])) OR ((("consensual"[All Fields] OR "consensually"[All Fields]) OR "consensus"[MeSH Terms]) OR "consensus"[All Fields])) OR (("guideline"[Publication Type] OR "guidelines as topic"[MeSH Terms]) OR "guidelines"[All Fields]))) AND **2019/6/27:2022/4/1[Date - Publication]**	1
#52	Search (((((Fetal monitoring) OR Cardiotocography)) AND (((((pregnancy) OR labor) OR delivery) OR child birth) OR birth))) AND (((((pathway) OR algorithms) OR clinical protocol) OR consensus) OR guidelines) Filters: Guideline	92
#51	Search (((((Fetal monitoring) OR Cardiotocography)) AND (((((pregnancy) OR labor) OR delivery) OR child birth) OR birth))) AND (((((pathway) OR algorithms) OR clinical protocol) OR consensus) OR guidelines)	1419

#50	Search ((((pathway) OR algorithms) OR clinical protocol) OR consensus) OR guidelines	1757129
#49	Search guidelines	401106
#48	Search consensus	164787
#47	Search clinical protocol	283717
#46	Search algorithms	341294
#45	Search pathway	647445
#44	Search (((Fetal monitoring) OR Cardiotocography)) AND (((((pregnancy) OR labor) OR delivery) OR child birth) OR birth)	15055
#43	Search ((((pregnancy) OR labor) OR delivery) OR child birth) OR birth	2557862
#42	Search (Fetal monitoring) OR Cardiotocography	16729
#41	Search birth	344227
#40	Search child birth	82414
#39	Search delivery	577012
#38	Search labour	1164806
#37	Search labor	1164806
#36	Search pregnancy	942502
#35	Search Cardiotocography	2667
#34	Search Fetal monitoring	16370
#33	Search Fetal monitoring Filters: Guideline	93

Database: Embase Initial search on June 27, 2019 and update on July 19, 2020

Embase Session Results

No.	Query	Results
#12	#10 AND #11	137
#11	#7 AND #8 AND [embase]/lim	1,686
#10	guideline:ti OR 'practice guideline':ti OR guidelines:ti	100,541
#9	#7 AND #8	2,000
#8	pathway OR 'algorithms'/exp OR algorithms OR 'clinical protocol'/exp OR 'clinical protocol' OR (('clinical'/exp OR clinical) AND ('protocol'/exp OR protocol)) OR 'consensus'/exp OR consensus OR 'practice guideline'/exp OR 'practice guideline'	2,216,847
#7	#5 AND #6	22,256
#6	'pregnancy'/exp OR pregnancy OR 'labor'/exp OR labor OR 'labour'/exp OR labour OR 'delivery'/exp OR delivery OR 'child birth'/exp OR 'child birth' OR (('child'/exp OR child) AND ('birth'/exp OR birth)) OR 'birth'/exp OR birth	2,068,962
#5	#3 OR #4	31,350
#4	'ctg'/exp OR ctg OR 'cardiotocography'/exp OR cardiotocography	10,772
#3	'fetal monitoring'/exp OR 'fetal monitoring' OR (fetal AND ('monitoring'/exp OR monitoring))	25,726
#2	('child'/exp OR 'child' OR 'children' OR 'pediatrics'/exp OR 'community paediatrics' OR 'community pediatrics' OR 'paediatric aspect' OR 'paediatric care' OR 'paediatric educating' OR 'paediatric education' OR 'paediatric institute' OR 'paediatric internship' OR 'paediatric perspective' OR 'paediatric practice' OR 'paediatric research' OR 'paediatric service' OR 'paediatrics' OR 'paediatrics department' OR 'pediatric aspect' OR 'pediatric care' OR 'pediatric educating' OR 'pediatric education' OR 'pediatric institute' OR 'pediatric internship' OR 'pediatric practice' OR 'pediatric research' OR 'pediatric service' OR 'pediatrics' OR 'pediatrics department' OR 'pediatry' OR 'social pediatry' OR 'well baby clinic' OR paediatrics OR 'adolescent'/exp OR 'infant'/exp) AND 'asthma'/exp AND ('aminophylline'/exp OR 'theophylline'/exp) AND (2014:py OR 2015:py OR 2016:py OR 2017:py OR 2018:py OR 2019:py OR 2020;py) AND ([adolescent]/lim OR [child]/lim OR [infant]/lim OR [preschool]/lim OR [school]/lim OR [young adult]/lim) AND ('aminophylline'/de OR 'theophylline'/de) AND [17-4-2019]/sd	27
#1	('child'/exp OR 'child' OR 'children' OR 'pediatrics'/exp OR 'community paediatrics' OR 'community pediatrics' OR 'paediatric aspect' OR 'paediatric care' OR 'paediatric educating' OR 'paediatric education' OR 'paediatric institute' OR 'paediatric internship' OR 'paediatric perspective' OR 'paediatric practice' OR 'paediatric research' OR 'paediatric service' OR 'paediatrics' OR 'paediatrics department' OR 'pediatric aspect' OR 'pediatric care' OR 'pediatric educating' OR 'pediatric education' OR 'pediatric institute' OR 'pediatric internship' OR 'pediatric practice' OR 'pediatric research' OR 'pediatric service' OR 'pediatrics' OR 'pediatrics department' OR 'pediatry' OR 'social pediatry' OR 'well baby clinic' OR paediatrics OR 'adolescent'/exp OR 'infant'/exp) AND 'asthma'/exp AND ('aminophylline'/exp OR 'theophylline'/exp) AND (2014:py OR 2015:py OR 2016:py OR 2017:py OR 2018:py OR 2019:py OR 2020;py) AND ([adolescent]/lim OR [child]/lim OR [infant]/lim OR [preschool]/lim OR [school]/lim OR [young adult]/lim) AND ('aminophylline'/de OR 'theophylline'/de)	170
